# Visual extraction of individual patient data from published kaplan-meier survival plots using a web-based application offering different reconstruction methods (V-Exploder)

**DOI:** 10.1186/s12874-026-03036-3

**Published:** 2026-10-05

**Authors:** Fabian Acker, Lea Ruge, Maximilian Rost, Hanna Schulte, Sophie Heinzen, Friederike C. Althoff, Khouloud Kouidri, Jan Stratmann, Eva Herrmann, Martin Sebastian, Sebastian E. Koschade

**Affiliations:** 1 Department of Medicine 2, Hematology and Oncology, Goethe University Frankfurt, University Hospital, Frankfurt, Germany; 2 Department of Medicine 1, Hematology and Oncology, University Hospital Cologne, Cologne, Germany; 3 Division of Hematology and Medical Oncology, Weill Cornell Medicine, New York, NY 10065 USA; 4 Department of Medicine 5, University Hospital Saarland, Pulmonology, Homburg, Germany; 5 Institute for Biostatistics and Mathematical Modelling, Goethe University Frankfurt, Frankfurt, Germany; 6 Frankfurt Cancer Institute, Goethe University, Frankfurt, Germany

**Keywords:** Survival analysis, Individual patient data (IPD), Kaplan-Meier plot, IPD reconstruction, Visual extraction, Meta-analysis, Cost-effectiveness analysis, V-Exploder

## Abstract

**Background:**

Individual patient-level survival data (IPD) is required for various types of medical research, such as meta-analyses and cost-effectiveness analyses. As IPD from clinical trials are usually not publicly available, secondary data utilization relies on IPD reconstruction from published Kaplan-Meier (KM) plots. In general, the established toolchains for IPD reconstruction are cumbersome and rarely offer possibilities to assess reconstruction accuracy.

**Implementation:**

We have therefore developed V-Exploder, an academic, non-profit, data-local tool that integrates the complete workflow of IPD reconstruction into a single browser application (www.v-exploder.com). Implemented methods include the iterative algorithms described by Guyot et al. and Rogula et al., and a novel, direct method for the positioning of individual events and censorings. A key feature of V-Exploder is its immediate visual feedback: recreated KM graphs are continuously constructed and superimposed onto the original figure. This enables more precise IPD reconstruction than previously possible.

**Methods:**

We conducted a study to evaluate the IPD reconstruction performance of V-Exploder. Ten investigators each reconstructed data from four of a total of twelve figures, each showing the survival of two randomly generated cohorts (20, 50, 100, or 200 subjects in each cohort). Metrics frequently used in survival analysis were calculated for the original and reconstructed data. Median absolute errors (AE), their interquartile ranges (IQR), and root square mean errors (RMSE) are reported.

**Results:**

Survival metrics from reconstructed IPD showed high similarity to metrics calculated from the reference data across all datasets, investigators and used methods of IPD reconstruction. Regarding log HRs, median AE (IQR) & RMSE with the direct, Rogula, and Guyot method were 5.93 × 10^− 4^ (7.61 × 10^− 5^, 1.65 × 10^− 3^) & 6.26 × 10^− 3^, 5.28 × 10^− 4^ (3.21 × 10^− 5^, 2.59 × 10^− 3^) & 4.05 × 10^− 3^, and 4.17 × 10^− 3^ (1.84 × 10^− 3^, 1.96 × 10^− 2^) & 2.38 × 10^− 2^, respectively. IPD reconstruction validity and reliability were lower with the Guyot method compared to the other methods, especially when used on smaller datasets. However, errors were considered acceptable for most research applications regardless of the method used.

**Conclusion:**

V-Exploder allows IPD reconstruction from published KM plots with high accuracy and reliability. Given its user-friendly visual interface, V-Exploder will facilitate secondary analysis of survival data and help improving analytic rigor.

**Supplementary Information:**

The online version contains supplementary material available at https://doi.org/10.1186/s12874-026-03036-3.

## Background

Time-to-event endpoints from clinical trials, including overall survival and progression-free survival, are typically reported using medians and survival rates at prespecified timepoints together with a Kaplan-Meier (KM) plot as graphical representation. When survival is compared between groups, differences in median survival as well as hazard ratios (HR) derived from Cox proportional hazards models are usually reported. While these aggregated metrics generally help to quantify differences between groups, they are frequently found to be insufficient for secondary data analyses including cost-effectiveness analyses [1–3] or meta-analyses [4, 5].

In the context of cost-effectiveness analyses, an estimate of the difference in survival is required which cannot be determined from medians or HRs without introducing assumptions on the distribution of survival data [1]. While meta-analyses typically incorporate HRs, the underlying requirement of proportional hazards is not always met and seldom checked [6]. Furthermore, meta-analyses may need to be conducted on specific subgroups for which HRs may not have been reported [7]. Ideally, these issues are addressed by using individual patient-level data (IPD).

It should be emphasized that IPD allow for further modeling techniques besides the settings outlined above. These include (1) the calculation of restricted mean survival times in the context of non-proportional hazards, (2) the fitting of parametric survival distributions for data extrapolation, the simulation of hypothetical trials, or other uses, as well as (3) the reconstruction of unreported subgroups, a method referred to as KM subtraction [8]. Finally, (4) the impact of assumed informative censoring can be visualized [9]. However, IPD are usually not publicly available, limiting any secondary data analyses of published outcome data. The Cochrane Handbook for Systematic Reviews of Interventions therefore recommends to employ reconstructed, approximate IPD [4].

Over the years, several methods for reconstructing IPD from published KM plots have been developed [10–12]. Conceptually, these methods are based on digitizing the coordinates of the axis system and specifying each point at which the KM curve drops, indicating one or several events. In a second step, survival estimates at the specified time points are calculated geometrically. Finally, an iterative algorithm is used to generate an IPD dataset which matches as closely as possibly with the reconstructed survival estimates. For the algorithm described by Guyot and colleagues, in addition to the survival estimates, the investigator must also provide the risk table, i.e. the number of subjects at risk at each time interval, and, if available, the total number of events observed [10]. Modified versions of this algorithm have been implemented for statistics software like Stata (IPDFC) [13] and R (IPDfromKM*) *[12] and are widely used. Another method, published by Rogula and colleagues, also incorporates the position of tick marks indicating censored observations on the KM graph, which may yield more accurate results [11]. The Guyot algorithm, in contrast, estimates the times of censorings.

**Fig. 1:**
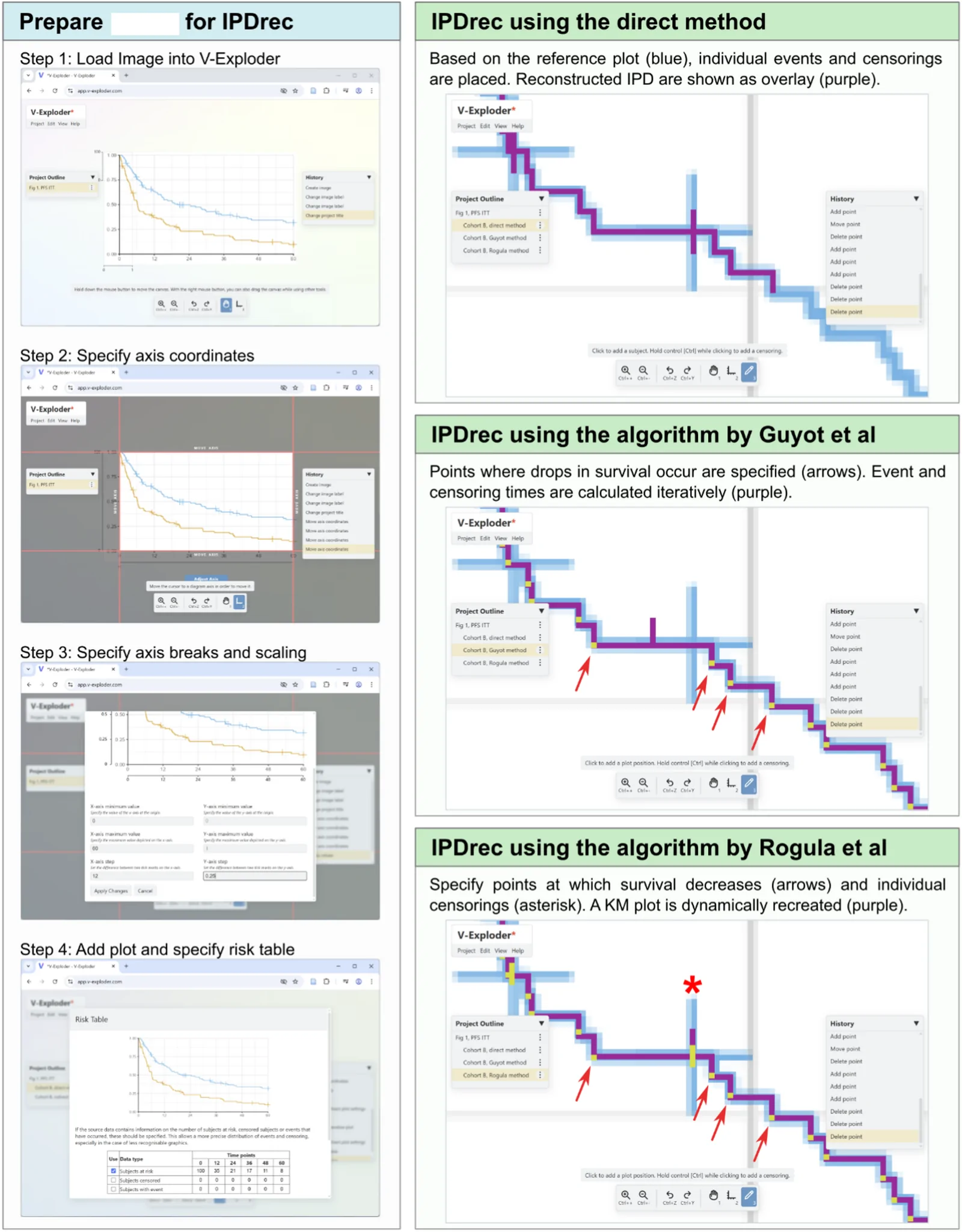
V-Exploder user interface and reconstruction workflow. IPDrec denotes individual patient survival data reconstruction.

Evidently, any secondary data use that incorporates approximate IPD is highly dependent on the accuracy of IPD reconstruction. The abovementioned reconstruction methods all do not implement the digitization step of the KM plots and require additional software to accomplish this. This leads to a workflow that is cumbersome and error-prone because it enables neither the assessment of reconstruction accuracy nor the correction of imprecisions until the final result is available [12]. To allow for more accurate and reliable IPD reconstruction, we have developed the Visual Extraction of Plot Data (V-Exploder) application. V-Exploder is an academic, non-profit, open-access tool that integrates the complete workflow of IPD reconstruction into a single portable browser application. A key feature of the app is its immediate visual feedback, as a KM curve is continuously generated from the reconstructed IPD and superimposed onto the original plot image. V-Exploder’s user interface and the workflow of IPD reconstruction are shown in Fig. 1. Here, we describe the app and report on the result of an evaluation study in which we compared IPD reconstructed with V-Exploder with the respective original data. A production-grade deployment of V-Exploder and an online documentation with a step-by-step guide is publicly available at www.v-exploder.com.

## Implementation

### Reconstruction methods

Technical aspects regarding the software architecture of V-Exploder are detailed in the Supplementary Appendix. The implemented methods of IPD reconstruction include the two abovementioned iterative algorithms described by Guyot et al. [10] and Rogula et al. [11]. Both algorithms have been re-implemented to run directly in the browser. This eliminates the requirement to upload (potentially confidential) data to a remote server. Re-implementations of both algorithms have been tested against their reference implementations, as described in the Supplementary Appendix.

Besides the iterative methods, a new method has been developed which we refer to as the *direct* method. With the direct method, individual events and censorings are set manually instead of specifying the coordinates of steps in the KM graph. This means that the investigator *directly* creates actual IPD without the need for an algorithm that generates IPD from survival estimates. As a consequence, the direct method provides maximum control over the reconstruction process. To our knowledge, this conceptually straightforward method has not been previously described in the literature, presumably because it requires a rich and dynamic user interface to yield accurate results.

### Reconstruction workflow

As illustrated in Fig. 1, the workflow of IPD reconstruction consists of several steps. First, an image file of the original KM plot is loaded into the app. The digital plot axes have to be aligned to match with the axes in the image and the investigator has to specify the maximum time and the time interval between axis breaks. After adding a new KM graph reconstruction (either for the direct or an iterative method), the investigator has to specify the data available in the figure’s subjects at risk table.

Next, the actual survival data are reconstructed. For the iterative methods, the investigator marks all points at which the KM graph drops and, in the case of the Rogula method, the positions of censored observations. When using the direct method, individual events and censorings are placed directly. If the superimposed, recreated KM graph does not match with the original figure, the investigator can modify or delete any previously set data.

Once the reconstructed KM graph is deemed sufficiently congruent to its original, IPD can be downloaded as a CSV file for further use in any statistical software.

### Data storage and safety

V-Exploder allows to handle multiple images, possibly with multiple KM graphs per image, within each project. A project file can be saved to disk to allow editing at a later time or to share reconstruction data for collaboration or external accuracy assessment. The file format (file extension “.vexploder”) includes a copy of the image files and was designed to enable backward compatibility. A technical description of the file format can be found in the Supplementary Appendix.

Although V-Exploder may be accessed via the web, all data related to IPD reconstruction are kept locally and are not uploaded to a remote server. This includes image files, data points, as well as the final IPD.

### Evaluation studies

We conducted an evaluation study to evaluate the IPD reconstruction performance of V-Exploder.

Random KM plots were generated as described in the Supplementary Appendix. Each plot contained two cohorts, both drawn from one of a total of three distributions (log-normal, log-logistic, and Weibull) and using one of a total of four different cohort sizes (20, 50, 100, 200 per cohort). Accordingly, the total number of figures was 12 with a total of 24 KM curves (Suppl. Figure 1 A). The figures had varying degrees of censoring and HRs between cohorts.

In addition, a publicly available real-world dataset from The Cancer Genome Atlas (TCGA) showing the survival of 200 patients with acute myeloid leukemia (AML) depending on age at disease onset, downloaded from www.cbioportal.com, was used (Suppl. Figure 1B) [14].

Ten investigators (including F.A. who developed the app) each reconstructed IPD for all four figures (each showing two cohorts with 20, 50, 100, and 200 subjects, respectively) of one of the three random survival distributions. Nine investigators reconstructed the TCGA AML dataset.

The investigators had varying levels of experience with IPD reconstruction. Most investigators reported having performed an IPD reconstruction either never (*n* = 7) or in 1–5 cases (*n* = 2). Only one investigator reported having reconstructed IPD from 50 or more plots. All investigators received a single introduction to the software, and a sample reconstruction was demonstrated.

### Statistics

For statistical analysis, all time data were scaled to 0–1 by setting the minimum observed time to 0 and the maximum observed time (T_max_) to 1. The scaling was performed at a figure-level, so that the survival of the two cohorts in a figure remained proportional.

We assessed the similarity between the reconstructed and original graphs for each cohort by calculating the absolute area *between* the curves as described in the Supplementary Appendix. In addition, we performed survival regression on each cohort using an exponential distribution and obtained its regression parameter (lambda).

Next, survival metrics commonly used in survival analysis were calculated for each reconstructed cohort or figure as well as using the reference IPD. On a cohort level, these metrics included 25%, 50% (median survival), and 75% survival quantiles and landmark survival at 60%, 80% and 100% of T_max_. On a figure level, log-transformed HRs and their uncertainties (standard error [SE] of the log HR) were calculated between the figure’s two cohorts.

Survival and similarity metrics calculated from reconstructed IPD were compared to those calculated with the reference data. Root mean square errors (RMSE, formula 1) and absolute errors (AEs, formula 2) were calculated to assess IPD reconstruction validity. Moreover, interquartile ranges (IQR) of AEs are provided, offering a measure of IPD reconstruction reliability across datasets and investigators.

Formula 1 – Calculation of root mean square errors (RMSE)

$$\:RMSE=\sqrt{\frac{1}{n}{\sum\:}_{i=1}^{n}{\left({y}_{i}-\widehat{{y}_{i}}\right)}^{2}}$$

Formula 2 – Calculation of absolute errors (AE)

$$\:AE=\:\frac{1}{n}\sum\:_{i=1}^{n}\left|{y}_{i}-{\widehat{y}}_{i}\right|$$

Comparing a self-calculated HR with the published HR is one of few ways to assess IPD reconstruction accuracy in practice. Since HRs are often reported with two or three decimal places, we calculated the proportion of cases with an AE in the untransformed HR below 0.01 or 0.001.

Finally, we examined whether the used IPD reconstruction methods performed differently in terms of the analyzed similarity and survival metrics, hypothesizing that all methods would yield similar results. We used Wilcoxon signed-tank tests on the AEs in the analyzed metrics across all datasets grouped by the used IPD reconstruction method. P-values were adjusted for multiplicity using the Bonferroni method. An adjusted p-value below 0.05 was considered significant.

## Results

**Fig. 2:**
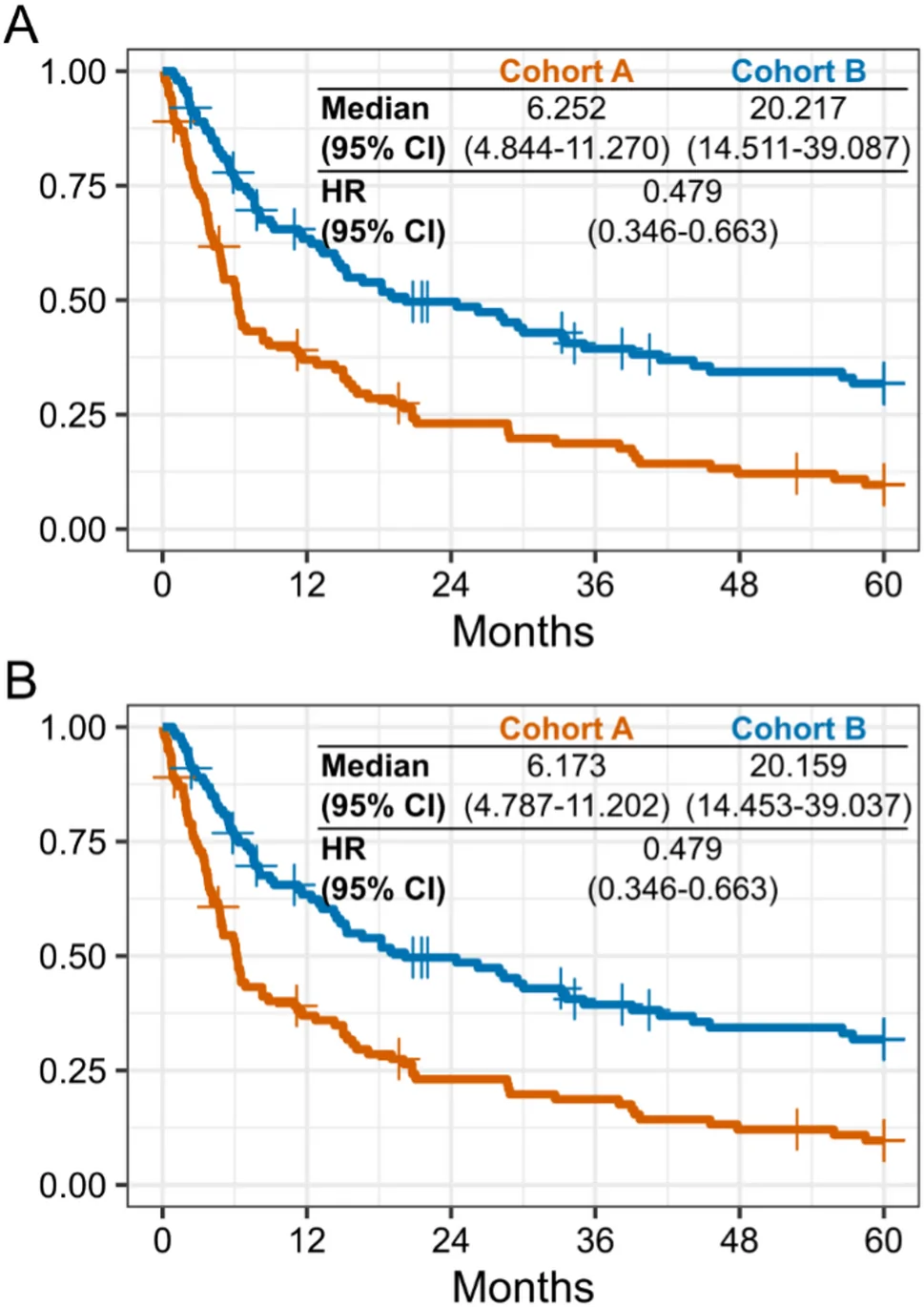
Example of KM plots from original and reconstructed IPD **A** Original IPD: Survival data were randomly drawn from a log-normal distribution with 200 subjects per cohort. **B** Reconstructed IPD: IPD were reconstructed using the direct method. After normalizing Tmax from 60 months to 1, the areas between the original and the reconstructed KM plots were 8.5×10-4 for cohort A and 6.1×10-4 for cohort B. CI denotes confidence interval, HR hazard ratio.

**Fig. 3:**
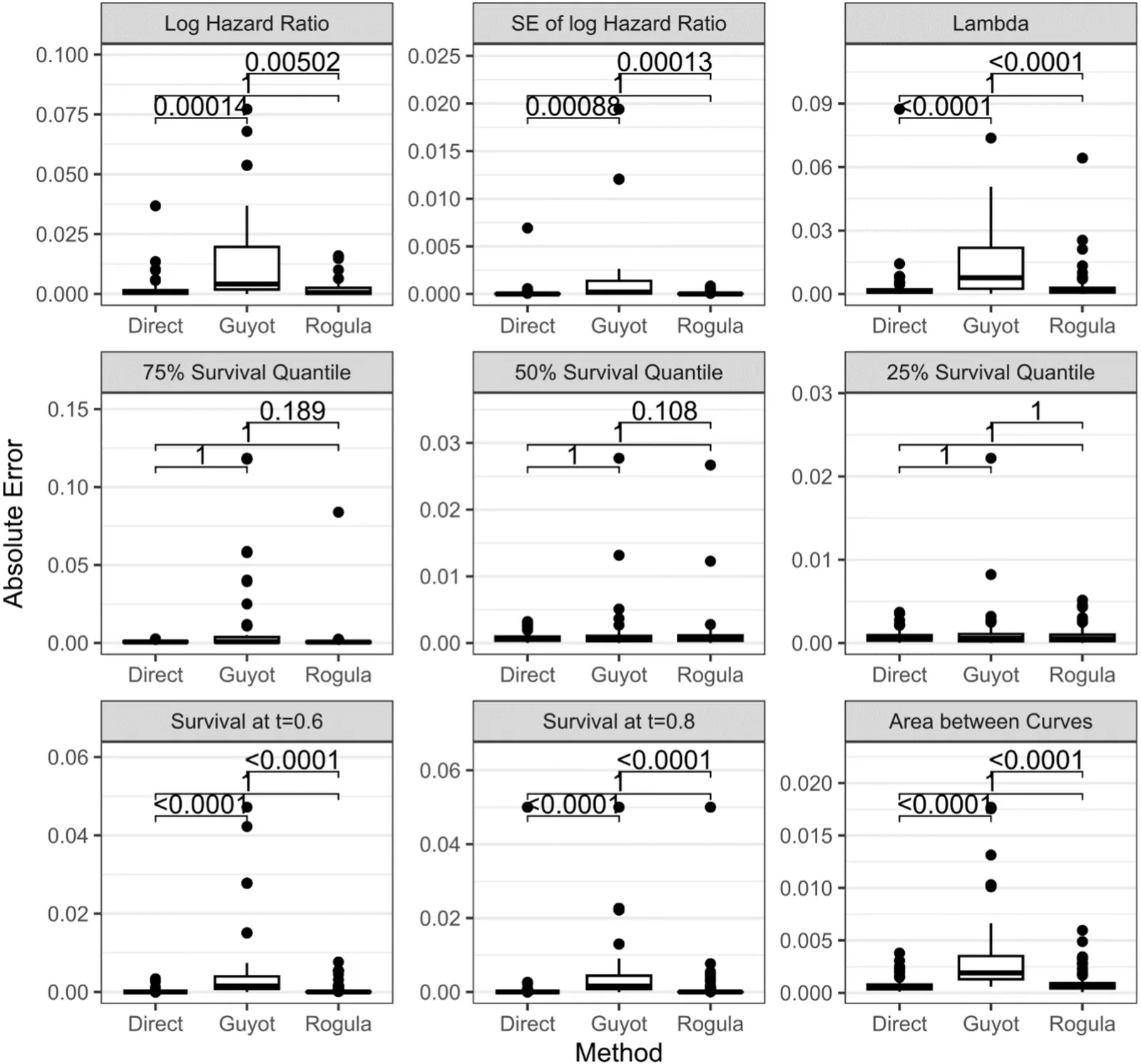
Absolute errors in survival metrics using reconstructed IPD by reconstruction method. Absolute errors in the analyzed metrics between reconstructed and original data are shown by the used reconstruction method and across all datasets. Lambda refers to the regression parameter of an exponential survival regression. P-values were obtained from Wilcoxon signed-rank tests and adjusted for multiplicity using the Bonferroni method. SE denotes standard error.

By visual inspection, KM plots from reconstructed IPD were found to be highly congruent with their original counterparts (Fig. 2). Regarding areas between curves, the median AE (IQR) was 7.93 × 10^− 4^ (5.05 × 10^− 4^, 1.76 × 10^− 3^) across all datasets, investigators and reconstruction methods. Using the direct, Guyot, or Rogula method, the median AE (IQR) in the areas between curves was 5.55 × 10^− 4^ (3.33 × 10^− 4^, 7.85 × 10^− 4^), 1.90 × 10^− 3^ (1.30 × 10^− 3^, 3.51 × 10^− 3^), and 6.19 × 10^− 4^ (4.25 × 10^− 4^, 9.17 × 10^− 4^), respectively (Table 1; Fig. 3). When performing survival regression, the median AEs (IQR) in the lambda parameter when using the direct, Guyot, or Rogula method was 1.36 × 10^− 3^ (6.75 × 10^− 4^, 2.15 × 10^− 3^), 7.67 × 10^− 3^ (2.47 × 10^− 3^, 2.18 × 10^− 2^), and 1.49 × 10^− 3^ (7.08 × 10^− 4^, 2.94 × 10^− 3^), respectively (Table 1; Fig. 3).

**Table 1:** Errors in survival metrics between original and reconstructed IPDSurvival metrics including hazard ratios between cohorts of a figure and the area between the Kaplan-Meier curves per cohort were calculated. Exponential survival regression was performed per cohort and its regression parameter (lambda) was obtained. Root mean square errors (RMSE, unit: 10-3) and absolute errors (unit: 10-3) between metrics in the reconstructed and the original datasets were calculated. For absolute errors, the median (50% AE) and interquartile ranges (25% AE, 75% AE) are reported. IPD denotes individual patient data, SE standard error.

Metric	Dataset(s)		No. distinctfigures	No. IPDsets	Errors by IPD reconstruction method (unit: 10^-3^)											
					Direct				Guyot				Rogula			
	Type	Size			25% AE	50% AE	75% AE	RMSE	25% AE	50% AE	75% AE	RMSE	25% AE	50% AE	75% AE	RMSE
Log hazard ratio	Random	20/arm	3	10	0.00	0.00	5.87	12.07	13.62	36.86	53.73	41.73	0.00	0.00	0.00	2.62
		50/arm	3	10	0.08	0.51	1.20	4.34	13.19	20.01	26.15	30.29	0.03	0.30	0.67	0.56
		100/arm	3	10	0.33	0.59	0.64	0.56	1.57	3.97	14.44	9.88	0.12	0.47	0.82	0.73
		200/arm	3	10	0.80	2.02	5.12	5.08	0.72	2.04	3.64	3.40	2.67	3.63	4.97	4.85
	TCGA	200 total	1	9	0.22	0.51	0.77	1.12	2.20	2.39	2.84	2.61	0.46	0.65	3.45	7.40
	Overall	-	13	49	0.08	0.59	1.65	6.26	1.84	4.17	19.64	23.80	0.03	0.53	2.59	4.05
SE log hazard ratio	Random	20/arm	3	10	0.00	0.00	0.25	2.19	0.64	1.80	12.05	9.07	0.00	0.00	0.00	0.11
		50/arm	3	10	<0.01	<0.01	0.02	0.18	0.18	0.43	1.65	1.25	<0.01	<0.01	0.01	0.01
		100/arm	3	10	<0.01	<0.01	<0.01	<0.01	0.04	0.18	0.45	0.69	<0.01	<0.01	<0.01	0.17
		200/arm	3	10	<0.01	0.06	0.15	0.11	0.01	0.02	0.21	0.17	0.02	0.02	0.14	0.10
	TCGA	200 total	1	9	<0.01	<0.01	<0.01	0.03	0.13	0.13	0.14	0.14	<0.01	0.02	0.03	0.30
	Overall	-	13	49	<0.01	<0.01	0.02	1.00	0.05	0.19	1.38	4.15	<0.01	<0.01	0.02	0.16
Area betweenthe curves	Random	20/arm	3	20	0.52	0.68	0.79	1.27	1.18	6.54	11.02	9.69	0.56	0.62	0.78	1.12
		50/arm	3	20	0.38	0.61	0.80	0.68	1.80	2.81	3.70	3.26	0.39	0.59	0.70	0.66
		100/arm	3	20	0.49	0.52	0.64	0.90	1.07	1.23	2.26	2.17	0.50	0.60	0.76	1.15
		200/arm	3	20	0.46	0.73	1.22	1.19	1.16	1.54	2.00	1.77	0.64	0.96	1.76	1.56
	TCGA	200 total	1	18	0.17	0.25	0.33	0.49	1.34	2.19	3.10	2.53	0.18	0.28	1.23	2.13
	Overall	-	13	98	0.33	0.55	0.79	0.96	1.30	1.90	3.51	4.91	0.43	0.62	0.92	1.39
Exponential survival regression (lambda)	Random	20/arm	3	20	1.22	1.45	2.32	19.73	3.03	11.71	21.36	22.57	1.03	1.48	1.98	2.57
		50/arm	3	20	0.47	0.95	1.84	1.60	7.82	13.75	19.14	24.58	0.55	0.86	1.78	1.71
		100/arm	3	20	1.09	1.44	2.14	3.78	1.33	2.60	4.62	6.66	1.02	1.51	1.96	3.53
		200/arm	3	20	1.30	2.06	4.27	3.65	1.29	2.51	3.16	2.69	1.16	2.37	3.60	3.05
	TCGA	200 total	1	18	0.27	0.60	1.23	2.29	26.01	28.02	28.71	27.57	0.39	2.38	6.98	17.44
	Overall	-	13	98	0.67	1.36	2.15	9.30	2.47	7.67	21.84	19.43	0.71	1.49	2.94	7.89

**Fig. 4:**
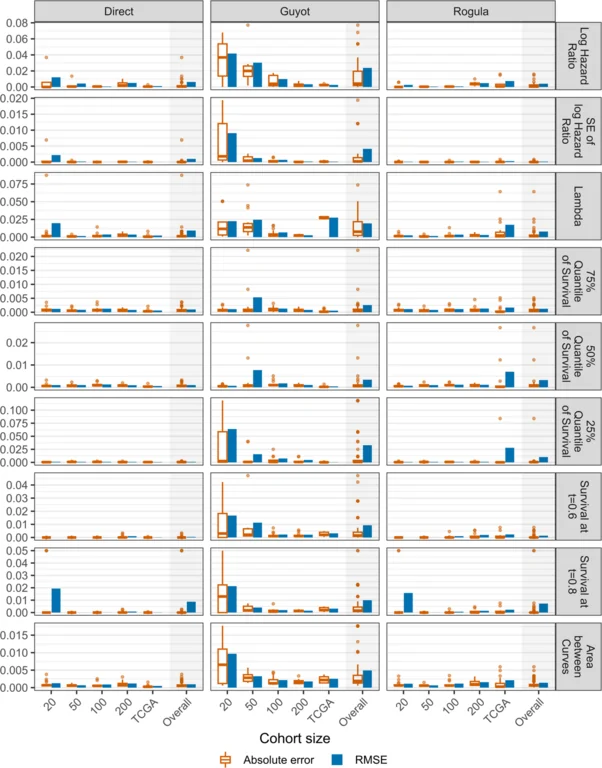
Errors in survival metrics using reconstructed IPD by method and cohort size. Absolute errors (box plots) in the analyzed metrics between reconstructed and original data are shown together with the respective root mean square errors (RMSE, bars). Data are grouped by the used IPD reconstruction method and sample sizes. Ten investigators each reconstructed IPD from four randomly generated figures (with 20, 50, 100, and 200 subjects per arm) showing survival of one of the three random distributions (Weibull, log-normal, log-logistic). The real-world TCGA AML dataset (200 subjects) was reconstructed 9 times. Lambda refers to the regression parameter of an exponential survival regression. Survival time is normalized within each figure (tmax=1). HR denotes hazard ratio, SE standard error.

Across datasets and investigators, IPD reconstructed using the Guyot method showed larger AEs in both the areas between curves and the lambda parameter compared with the other methods (P-values each < 0.0001). In contrast, no significant differences were observed between the direct method and the Rogula method (Fig. 3). When looking at the errors by cohort size, errors in metrics using Guyot-derived IPD were particularly high when the method was employed on smaller datasets (Fig. 4).

Survival metrics calculated from reconstructed IPD generally showed high similarity to those calculated using the reference data (Table 1; Fig. 3). Regarding log-transformed HRs, the observed errors in datasets obtained using the direct, Guyot, and Rogula method were generally small with a median AE (IQR) across all datasets and investigators of 5.93 × 10^− 4^ (7.61 × 10^− 5^, 1.65 × 10^− 3^), 4.17 × 10^− 3^ (1.84 × 10^− 3^, 1.96 × 10^− 2^), and 5.28 × 10^− 4^ (3.21 × 10^− 5^, 2.59 × 10^− 3^), respectively (Table 1; Fig. 3). RMSEs of the log HR were 6.26 × 10^− 3^, 2.38 × 10^− 2^, and 4.05 × 10^− 3^, respectively.

Log HRs calculated from IPD reconstructed using the Guyot method had larger AEs as those from either the direct or the Rogula method, indicating both lower IPD reconstruction validity and reliability compared with the direct and Rogula method (Fig. 3). Again, this was particularly apparent when the Guyot method was employed on smaller cohorts, whereas errors seemed to converge with those from the other methods when the Guyot method was used on larger datasets (Fig. 4).

Of HRs calculated from direct and Rogula IPD, 94% (95% CI, 83–99) and 89% (78–97) had an AE smaller than 0.01, and 59% (44–73) and 59% (44–73) had an AE smaller than 0.001, respectively. In contrast, only 32% (95% CI, 50–78) and 18% (9–32) of HRs calculated with IPD estimated using the Guyot method had an absolute error less than 0.01 or 0.001, respectively.

The uncertainties of HRs, measured as SE of log HR, were higher with Guyot-derived data (median AE, 1.92 × 10^− 4^; IQR 4.75 × 10^− 5^, 1.38 × 10^− 3^) than with data from the direct (4.50 × 10^− 6^; 5.20 × 10^− 7^, 2.34 × 10^− 5^) and the Rogula method (7.32 × 10^− 6^; 2.07 × 10^− 6^, 2.22 × 10^− 5^; Table 1; Fig. 3).

Survival quantiles from reconstructed IPD, including medians, generally showed very high similarity with their respective references (Fig. 3; Table 1, Suppl. Table 1). Errors in landmark survival were generally low across the used reconstruction methods but were slightly higher with data reconstructed using the Guyot method (Fig. 3; Table 1). As with the similarity metrics and HRs, survival quantiles and landmark survival using Guyot-derived data showed a tendency towards higher errors in the context of smaller datasets (Fig. 4; Table 1).

## Discussion

IPD is essential for various types of secondary data use in medical research. Consequently, reliable and validated IPD reconstruction tools are needed. The workflows proposed so far are based on a three-step process of digitizing the images, then preparing the data for the algorithms and, finally, executing them. This process does not allow to compare the congruency of the reconstructed KM curve with the original curve during reconstruction. We hypothesized that a more accurate IPD reconstruction can be achieved by implementing an integrated workflow that provides direct feedback during reconstruction through ad-hoc visualization. Therefore, we have developed a cross-platform browser app for the reconstruction of IPD that implements two established iterative algorithms and a novel direct method with a user-friendly interface and the possibility to save data for later use and collaboration.

In our evaluation study, both similarity metrics and survival metrics calculated from reconstructed IPD showed high congruency with the respective reference values across investigators and datasets. Over 90% of HRs calculated with IPD from the direct or Rogula method had an AE smaller than 0.01. In approximately 60% of cases, the AE was smaller than 0.001. These thresholds correspond to the limit of detecting differences when comparing with published HRs, as HRs are typically reported with two or three decimal places. We considered the overall accuracy of reconstructed IPD to be sufficient for all common types of secondary data use.

We observed that errors were generally higher for analyses based on Guyot-derived data as compared with data obtained using the other reconstruction methods. This is presumably caused by the algorithm’s tendency to accumulate errors in survival estimates due to less accurate placement of censorings. This sometimes results in visible deviations of the reconstructed and original KM graphs at the tail of the curve, particularly with smaller datasets, as described previously [11]. In our study, this is reflected by the high errors in landmark survival at t = 0.6 and t = 0.8. Although the errors may still be acceptable for most applications, the direct and Rogula methods should be prioritized in scenarios where such metrics are of interest.

The original publications of Guyot et al. and Rogula et al. also reported on evaluation studies of their respective algorithms and the associated workflows, but only Guyot et al. published mean absolute errors of survival metrics [10, 11]. Although we used different synthetic and real-world datasets in our validation study, we observed similar mean AEs (Suppl. Table 2) when comparing our results obtained using the Guyot method in V-Exploder to those Guyot et al. reported in their work, corroborating our study. Importantly, we were able to further improve the reconstruction accuracy of these datasets by using the algorithm by Rogula et al. as well as our novel *direct* method.

Several limitations to IPD reconstruction in general and in our study remain. First, the image quality of the underlying figure can significantly influence how easily its data can be reconstructed. The images used in our study were of good quality, and we decided not to conduct an evaluation using poor-quality images, as this would have been difficult to standardize.

Another potential issue is the degree of censoring. When the censoring indicators in the image begin to overlap, the direct method and the Rogula method may be more difficult to apply. In our experience, both methods remain usable to some extent, but may require some tweaking in order to identify the correct sequence of events and censored observations. In cases of excessive censoring, such as in large-scale registry data, the Guyot algorithm, which estimates censoring, may be a useful and time-saving alternative. Of note, the Guyot method also provides the quickest results as it requires the least input from the user. However, it is unclear whether the Guyot method actually performs better than the direct method or the Rogula method in the setting of excessive censoring. In our study, the degree of censoring in the synthetic cohorts ranged from 0% to 68% (Suppl. Figure 1 A), but we did not formally assess the impact of censoring on reconstruction accuracy.

Modified versions of the algorithm by Guyot et al. have been implemented in the Stata package IPDFC and the R package IPDfromKM [12, 13]. Both packages require a list of survival estimates, the times they were captured at, and the risk table data as inputs. The user has to transform pixel coordinates into survival estimates or time values before calling the packages’ reconstruction functions. For IPDfromKM, an online application is available that offers a full workflow consisting of two steps. In the first step, the user can digitize survival estimates and download a list of time-survival pairs. In the second step, the list can be uploaded for the actual IPD reconstruction. Although the IPDfromKM application offers functionalities for comparing the provided survival estimators with the ultimately reconstructed IPD, there is no way to modify the user inputs at this stage and digitization has to be restarted. In contrast, V-Exploder continuously displays generated KM graphs on top of the original figure and allows modification of any input at any time. In addition, V-Exploder project files can be saved for later re-use and distribution which may ultimately help improving scientific rigor in IPD reconstruction.

Finally, we would like to point out, that neither of the mentioned reconstruction methods is suitable for reconstructing survival data from Aalen-Johansen plots, which are frequently used to visualize survival in multi-state survival or competing risk models [15], or Simon-Makuch plots, which are used in the setting of time-varying variables [16]. Consequently, V-Exploder does not support IPD reconstruction for either of these plots.

## Conclusions

V-Exploder integrates the entire workflow of reconstructing IPD from published KM plots in a single web application. It produces reliable and accurate results. With its user interface that offers immediate visual feedback and the ability to save project files for later use, it will facilitate secondary data use in medical research such as meta-analysis or cost-effectiveness analyses.

**Availability and requirements**.

- Project name: V-Exploder.
- Project home page: https://www.v-exploder.com.
- Operating system(s): platform-independent.
- Programming language: TypeScript.
- Other requirements: none.
- License: custom license (see source files).
- Any restrictions to use by non-academics: see terms of use (see source files).

## Supplementary information

Below is the link to the electronic supplementary material.

Supplementary Material 1

Supplementary Material 2

## Data Availability

The V-Exploder source files can be found in the supplementary appendix. The datasets used and/or analyzed during the current study are available from the corresponding author on reasonable request.

## Abbreviations

AE: Absolute error
AML: Acute myeloid leukemia
HR: Hazard ratio
IPD: Individual patient data
IQR: Interquartile range
KM: Kaplan-Meier
RMSE: Root mean square error
SE: Standard error
T_max_: Maximum observed time
