# Supplementary material for: Visual extraction of individual patient data from published kaplan-meier survival plots using a web-based application offering different reconstruction methods (V-Exploder)
Source: BMC Med Res Methodol. 2026 Oct 5;26:216. doi: 10.1186/s12874-026-03036-3 (PMC13640098; doi:10.1186/s12874-026-03036-3)
Supplement: Supplementary file 1 [file 12874_2026_3036_MOESM1_ESM.pdf]

Supplementary Appendix

Article

Visual Extraction of Individual Patient Data from Published Kaplan-Meier Survival Plots using a Web-based Application (V-Exploder)

Corresponding Author

Dr. Fabian Acker, Goethe University Frankfurt, University Hospital, Department of Medicine II, Hematology and Oncology, Theodor-Stern-Kai 7, 60590 Frankfurt, Germany, e-mail: acker@med.uni-frankfurt.de

Table of Contents

Supplementary Appendix 1

- Technical aspects 2
- Implementation equivalence of the iterative algorithms Guyot and Rogula 2
- Description of the .vexploder file format 2
- Creating survival plots for the evaluation study 4
- Calculating the areas between Kaplan-Meier curves 6
- Suppl. Figure 1A 7
- Suppl. Figure 1B 8
- Suppl. Table 1 9
- Suppl. Table 2 11

Technical aspects

V-Exploder is written in TypeScript, a superset of JavaScript, which is transpiled into JavaScript during the build process and can therefore be executed natively in the browser. The app is built on a modular platform providing only the graphical user interface and associated basic functionalities including a history of user commands, which enables implementing undoing/redoing commands. Further app features are realized within modules. For example, there are modules for the management of image files and plot metadata. There are currently two modules implementing IPD reconstruction. The "km" module implements the direct method, the "kmn" module implements the iterative algorithms by Guyot et al. and Rogula et al.

Implementation equivalence of the iterative algorithms Guyot and Rogula

The V-Exploder source code is transpiled into JavaScript and, thus, can be executed natively in the browser. The reference implementations of the iterative algorithms described by Guyot and colleagues and Rogula and colleagues are written in R. It is worth noting that JavaScript and R follow fundamentally different concepts and that code in one language is comparatively difficult to translate into the other.

However, to allow for prompt visual feedback, i.e. the display of reconstructed Kaplan-Meier (KM) plots as overlay over the original figures, the iterative algorithms by Guyot and Rogula had to be reimplemented in TypeScript. The reference implementations provided in the original publications were translated expression by expression. In the case of the Guyot algorithm, additional code was required to transform V-Exploder data (i.e. pixel coordinates of survival estimates) into the algorithm's actual input.

Unit tests have been created to test the V-Exploder implementations with the reference implementations. During these tests, a random number of synthetic subjects are drawn from an exponential distribution with randomly chosen lambda and a randomly chosen censoring rate (0-1). The inputs required for the iterative algorithms are then calculated. This includes the risk table for a fixed time interval between axis breaks as well as the "true" relative coordinates of steps in a hypothetical KM plot. The respective data are then used to invoke both the original implementations in R as well as the TypeScript-based custom implementations. Results are compared while accounting for different standards between R and JavaScript regarding floating-point imprecisions.

Of note, these tests are not deterministic (i.e., they make heavy use of pseudo-random numbers). After debugging of the custom implementations was considered finalized, they were repeated several 100.000 times without showing any deviation in results of the original and the V-Exploder implementations of both the Guyot and the Rogula algorithm.

Description of the .vexploder file format

A V-Exploder file (extension ".vexploder") consists of several chunks containing different types of data. Chunks are stored one after each other without any offset. Except for the "start" chunk that stands at the top of every V-Exploder file, every chunk starts with a 4

bytes long chunk type identifier followed by a 4-byte unsigned integer specifying the size of the chunk in bytes. The file must start with the “start” chunk.

Chunk type	Variable	Type	Description
Start	Header	Ascii[8]	Must equal “vexplodr”
Start	File format version	Uint32	File format version
Start	Human-readable file description	Ascii[64]	May be any string. Contains a human-readable reference to the V-Exploder website
Project	Chunk type	Ascii[4]	Must equal “proj”
Project	Chunk size	Uint32	-
Project	Project title	Utf16	Human-readable title of the project. The chunk size specifies the size of this string in bytes
Image	Chunk type	Ascii[4]	Must equal “imgs”
Image	Chunk size	Uint32	-
Image	Image id	Uint32	-
Image	Image label size (bytes)	Uint32	-
Image	coordOriginX	Float32	X-coordinate in pixels of the axis origin in this image, starting from the top left corner
Image	coordOriginY	Float32	Y-coordinate in pixels of the axis origin in this image, starting from the top left corner
Image	coordMaxX	Float32	X-coordinate in pixels of the image’s right end
Image	coordMaxY	Float32	Y-coordinate in pixels of the image’s top end
Image	valueOriginX	Float32	The X-value at the axis origin (should be 0)
Image	valueOriginY	Float32	The Y-value at the axis origin (should be 0)
Image	valueMaxX	Float32	The maximum value on the X-axis (T_{max})
Image	valueMaxY	Float32	The maximum value on the Y-axis (should be 1)
Image	numBreaksX	Uint32	Number of breaks on the X-axis
Image	numBreaksY	Uint32	Number of breaks on the Y-axis
Image	Image label	Utf16[]	The image label size in bytes is specified earlier
Image	MIME type size (bytes)	Uint32	The size of the MIME type specifier in bytes
Image	MIME type	Ascii[]	
Image	Image data	Byte[]	The size of the image data is specified earlier. This contains an actual image file (jpg, png, …)
Plot	Chunk type	Ascii[4]	Must equal “plot”
Plot	Chunk size	Uint32	-
Plot	Plot id	Uint32	-
Plot	Module name size (bytes)	Uint32	Size in bytes of the module name
Plot	Plot label size (bytes)	Uint32	Size in bytes of the plot label
Plot	Module name	Ascii[]	The V-Exploder module that is responsible for handling this plot. The “km” module handles KM data reconstructed using the direct method whereas the iterative methods have been implemented in the “kmn” module
Plot	Plot label	Utf16[]	-
KM	Chunk type	Ascii[4]	Must equal “km”. A KM chunk contains data from the “km” module, i.e. the IPD reconstruction data of the direct method
KM	Chunk size	Uint32	-
KM	Plot id	Uint32	The id of the plot this km data belongs to
KM	Number of subjects	Uint32	-
KM	Number of table entries	Uint32	Table refers to the at-risk table
KM	Risk table flags	Uint32	Bitmask (1=atRisk, 2=censored, 4=events)
KM	Color string size	Uint8	-
KM	Color string	Ascii[]	HTML/CSS-valid color identifier, e.g. “#000000” or “red”
KM	Subjects	Subject[]	As follows:
KM.Subject	coordX	Float32	X-coordinate (in pixels) in the image of this event/censoring
KM.Subject	Status	Uint8	Either 1 (event) or 0 (censored)
KM	Table entries	Entry[]	As follows:

Chunk type	Variable	Type	Description
KM.Entry	Break	Uint32	The number of the break on the X-axis, this entry belongs to (0=origin)
KM.Entry	At risk	Uint32	No. subjects at risk
KM.Entry	Censored	Uint32	No. subjects censored
KM.Entry	Events	Uint32	No. subjects with event
KMN	Chunk type	Ascii[4]	Must equal “kmn”. AN KM chunk contains data from the “kmn” module, i.e. the IPD reconstruction data of an iterative method (Guyot or Rogula)
KMN	Chunk size	Uint32	-
KMN	Plot id	Uint32	The id of the plot this km data belongs to
KMN	Number of points	Uint32	-
KMN	Number of table entries	Uint32	Table refers to the at-risk table
KMN	Whether the overall no. events is specified	Uint8	Boolean
KMN	The overall number of events	Uint32	-
KMN	Method	Uint8	1=Guyot, 2=Rogula
KMN	Plot color string size	Uint8	-
KMN	Point color string size	Uint8	-
KMN	Points	Point[]	As follows:
KMN.Point	coordX	Float32	X-coordinate (in pixels) in the image of this point
KMN.Point	cordy	Float32	Y-coordinate (in pixels) in the image of this point
KMN.Point	Type	Uint8	0=point, 1=censoring
KMN	Table entries	Entry[]	As follows:
KMN.Entry	Break	Uint32	The number of the break on the X-axis, this entry belongs to (0=origin)
KMN.Entry	At risk	Uint32	No. subjects at risk
KMN	Plot color string	Ascii[]	HTML/CSS-valid color identifier, e.g. “#000000” or “red”
KMN	Point color string	Ascii[]	HTML/CSS-valid color identifier, e.g. “#000000” or “red”

Creating survival plots for the evaluation study

This R code was used to create the synthetic survival data for the evaluation study. It was executed with R version 4.4.2 (2024-10-31 ucrt), platform: x86_64-w64-mingw32/x64, Windows 11 x64 (build 22631). Used packages were survsim 1.1.8, ggplot2 3.5.1, ggsurvfit 1.1.0, survival 3.7-0.

```
library("survsim")
library("survival")
library("ggsurvfit")

# settings
save = TRUE
folder = R.utils::getAbsolutePath("generated")

# uses survsim::simple.surv.sim() to generate survival data.
# if the global "save" variable is TRUE, this function will store a
# Kaplan-Meier plot and survival data in "*prefix*/*name*.(csv|png)".
generate_study = function(name, seed, n, dist.ev, anc.ev1, beta0.ev1, anc.ev2, beta0.ev2) {
  set.seed(seed)
  foltime=60
  cohort1 = simple.surv.sim(
    n=n, foltime=foltime,
    dist.ev=dist.ev, anc.ev=anc.ev1, beta0.ev=beta0.ev1,
    dist.cens="unif", anc.cens=foltime*5, beta0.cens=0
  );

  cohort2 = simple.surv.sim(
    n=n, foltime=foltime,
    dist.ev=dist.ev, anc.ev=anc.ev2, beta0.ev=beta0.ev2,
    dist.cens="unif", anc.cens=foltime*5, beta0.cens=0
```

```
  );

  cohort1$group = "Cohort A"
  cohort2$group = "Cohort B"
  data = rbind(cohort1, cohort2)
  df = data.frame(time=data$stop, event=data$status, group=data$group)

  fit = survfit2(Surv(time, event) ~ group, data=df)
  p = (
    ggsurvfit(fit, size=1, lineend="round", linejoin="round")
    + add_risktable(risktable_stats="n.risk")
    + add_censor_mark(size=2)
    + scale_x_continuous(breaks=seq(0, 60, 6))
    + scale_y_continuous(breaks=seq(0, 1, .1))
    + coord_cartesian(c(0, 60), c(0, 1), expand=T, clip="off")
    + theme(
      legend.position="bottom",
      title=element_text(size=14, color="#333"),
      axis.title=element_text(size=14, color="#333"),
      axis.text=element_text(size=14, color="#333"),
      panel.grid=element_blank()
    )
    + ggtitle(paste0('Random data "', name, '" (seed ', seed,')'))
  )

  if(save) {
    dpi=72
    ggsave(
      file.path(folder, paste0(name, ".png")),
      plot=p,
      width=1200*dpi/72, height=800*dpi/72, units="px", dpi=dpi
    );
    write.table(
      df,
      file=file.path(folder, paste0(name, ".csv")),
      quote=T, eol="\n", sep=";", dec=".", append=F,
      col.names=T, row.names=F,
    );
  }

  print(p)
}

generate_study("lnorm20", 64324984, 20, "lnorm", 1.012, 1.744, 0.721, 3.121)
generate_study("lnorm50", 54651931, 50, "lnorm", 1.121, 2.119, 0.7, 3.251)
generate_study("lnorm100", 89123115, 100, "lnorm", 1.542, 1.987, 1.675, 3.145)
generate_study("lnorm200", 15132133, 200, "lnorm", 1.394, 2.691, 1.432, 3.203)
generate_study("weibull20", 49732167, 20, "weibull", 0.928, 4.125, 0.658, 2.754)
generate_study("weibull50", 98251975, 50, "weibull", 0.928, 4.125, 0.658, 2.754)
generate_study("weibull100", 75729124, 100, "weibull", 1.328, 3.125, 1.058, 2.354)
generate_study("weibull200", 34984324, 200, "weibull", 1.128, 2.525, 1.658, 3.154)
generate_study("llogistic20", 19740531, 20, "llogistic", 0.763, 2.564, 0.514, 3.121)
generate_study("llogistic50", 44346890, 50, "llogistic", 0.804, 3.102, 0.432, 4.002)
generate_study("llogistic100", 89419871, 100, "llogistic", 0.646, 3.145, 0.550, 3.373)
generate_study("llogistic200", 14892764, 200, "llogistic", 0.701, 4.404, 0.731, 3.401)
```

Calculating the areas between Kaplan-Meier curves

The following R function was used to calculate the absolute areas between two Kaplan-Meier curves.

```
#' This function calculates the absolute area between two Kaplan-Meier curves.
#' This means that between every steps in the curves the absolute of the delta
#' survival will be multiplied with delta time and added.
#' Of note, this function also normalizes for the maximum observed time.
#'
#' a and b are data frames of KM data and have a time and a status column.
calcMetricAbsABC = function(a, b) {
  a.end = F # end of cohort a reached
  b.end = F
  a.i = 1 # current element in a
  b.i = 1
  a.S = 1 # current survival in a
  b.S = 1
  a.atRisk = nrow(a) # subjects left at risk in a
  b.atRisk = nrow(b)
  times = sort(unique(rbind(a$time, b$time)))
  t.last = 0
  ABC = 0 # resulting ABC

  # duplicate the last time in the list of times of steps in the KM plot.
  # with this, the loop does not miss the last part of the graph
  times = c(times, max(times))

  # traverse all steps in the KM curves
  for(t in c(times, max(times))) {
    # calc ABC between last time (t.last) and the current time (t)
    dt = t - t.last
    dS = abs(a.S - b.S)
    ABC = ABC + dS * dt

    # stop if either cohort is finished
    if(a.end || b.end)
      break;

    # proceed in a
    while(!a.end && a[a.i, "time"] <= t) {
      if(a[a.i, "status"] == 1)
        a.S = a.S * (a.atRisk - 1) / a.atRisk
      a.atRisk = a.atRisk-1
      a.i = a.i+1
      if(a.i >= nrow(a)) a.end = T
    }

    # proceed in b
    while(!b.end && b[b.i, "time"] <= t) {
      if(b[b.i, "status"] == 1)
        b.S = b.S * (b.atRisk - 1) / b.atRisk
      b.atRisk = b.atRisk-1
      b.i = b.i+1
      if(b.i >= nrow(b)) b.end = T
    }

    t.last = t;
  }

  ABC / t; # normalize
}
```

Suppl. Figure 1A

Kaplan-Meier plots of the randomly created datasets. Hazard ratios (HR) are shown together with the number of events by cohort (data maturity). Cohort A is shown in orange, cohort B in blue.

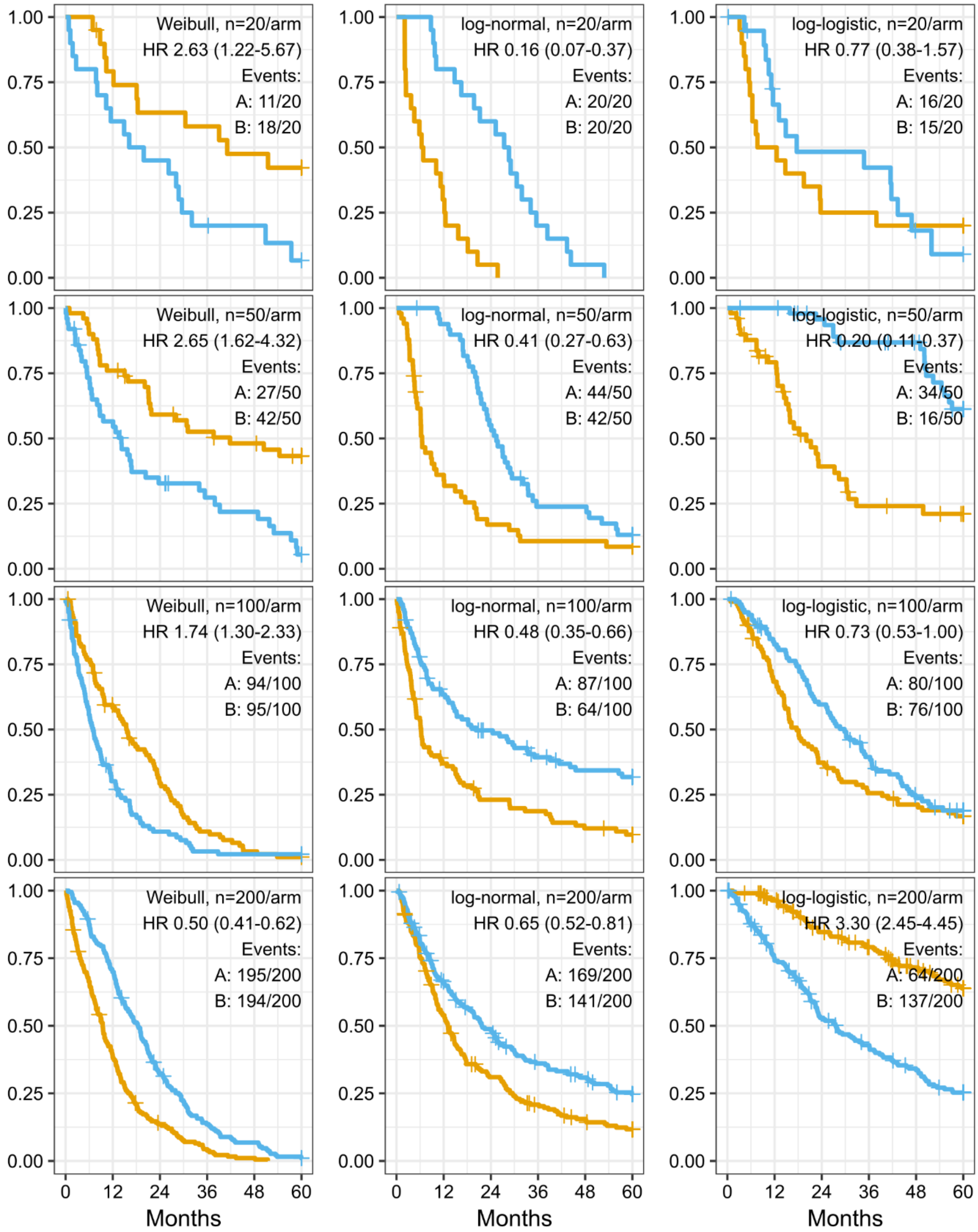

Suppl. Figure 1B

Kaplan-Meier plot of the real-world TCGA AML dataset

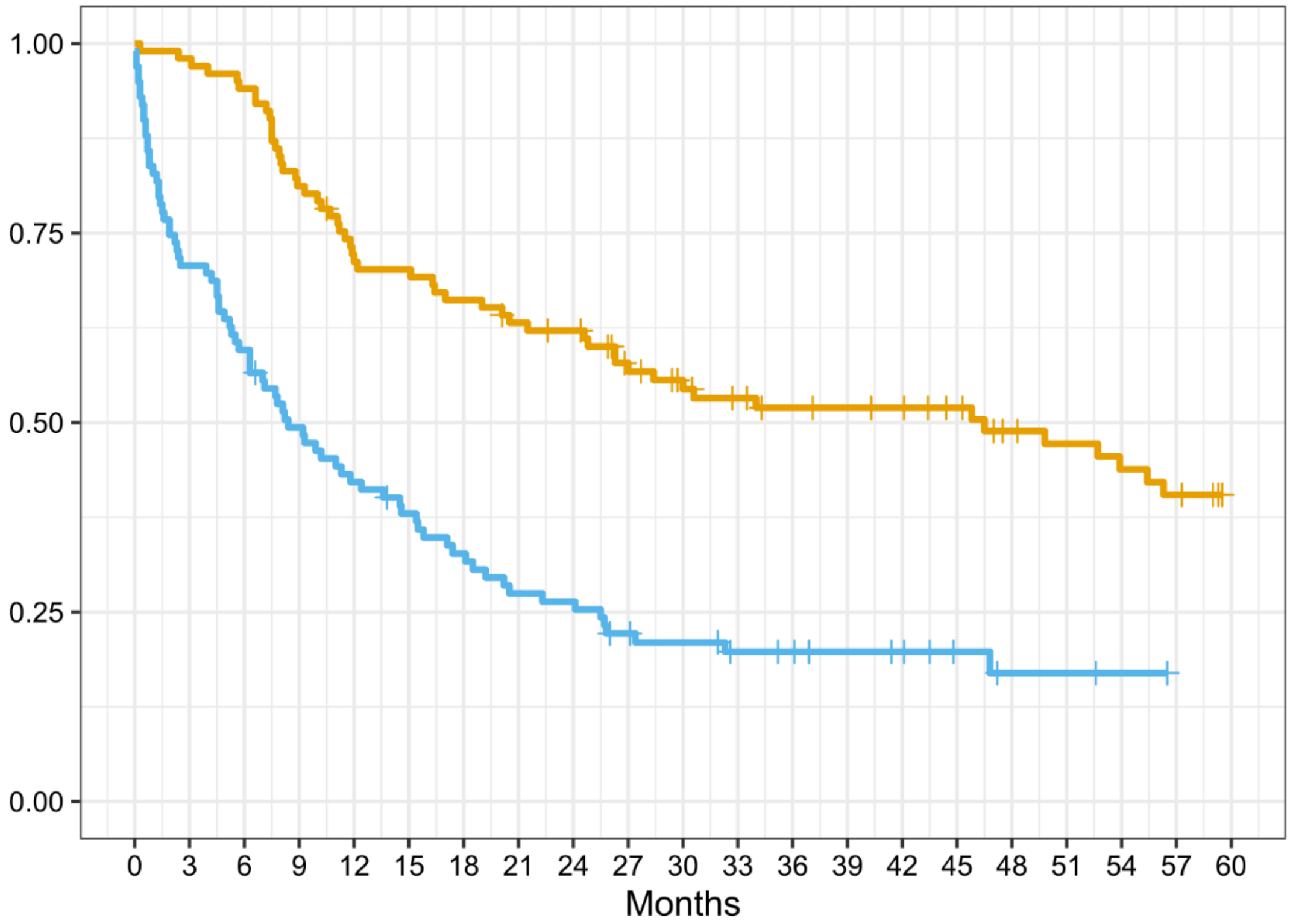

At Risk

	0	3	6	9	12	15	18	21	24	27	30	33	36	39	42	45	48	51	54	57	60
A, original	101	99	95	82	72	70	66	62	60	52	47	43	40	39	38	35	30	28	26	24	20
B, original	99	70	59	48	41	36	31	26	25	20	18	15	14	11	10	7	5	5	4	3	3

Suppl. Table 1

Hazard ratios between cohorts as well as cohort-level landmark survival and survival quantiles were calculated. Absolute errors (AE) and root mean square errors (RMSE) were calculated between metrics calculated on reconstructed data and on the original data (unit: 10^{-3}). For AEs, median (50% AE) and interquartile ranges (25% AE, 75% AE) are reported. IPD denotes individual patient data.

Metric	Dataset(s)		No. distinct figures	No. IPD sets	Errors by IPD reconstruction method (unit: 10^{-3})											
					Direct				Guyot				Rogula			
	Type	Size			25% AE	50% AE	75% AE	RMSE	25% AE	50% AE	75% AE	RMSE	25% AE	50% AE	75% AE	RMSE
Hazard ratio	Random	20/arm	3	10	0.00	0.00	0.94	30.05	10.78	40.33	84.03	58.84	0.00	0.00	0.00	0.42
		50/arm	3	10	0.03	0.19	2.52	2.01	2.71	11.50	46.24	31.29	0.03	0.18	0.32	0.92
		100/arm	3	10	0.22	0.47	0.64	0.61	0.87	1.91	22.67	16.17	0.06	0.37	0.69	1.02
		200/arm	3	10	0.99	1.89	6.03	12.28	1.77	2.25	2.75	2.76	1.64	4.25	11.28	9.40
	TCGA	200 total	1	9	0.58	1.35	2.06	2.97	5.85	6.36	7.57	6.94	1.23	1.74	9.18	19.57
	Overall	-	13	49	0.05	0.68	1.78	14.75	1.69	5.26	28.87	31.15	0.05	0.70	2.26	9.42
75% survival quantile	Random	20/arm	3	20	0.42	0.74	1.09	1.20	0.39	0.67	0.99	1.12	0.39	0.68	1.00	1.13
		50/arm	3	20	0.28	0.58	0.77	0.86	0.25	0.53	1.07	5.35	0.24	0.47	0.90	0.82
		100/arm	3	20	0.43	0.72	1.03	1.27	0.53	0.98	1.38	1.30	0.39	0.59	1.11	1.14
		200/arm	3	20	0.31	0.57	1.04	0.83	0.29	0.55	1.05	0.84	0.45	0.56	1.06	1.32
	TCGA	200 total	1	18	0.19	0.33	0.41	0.63	0.08	0.17	0.38	0.56	0.08	0.16	0.41	1.68
	Overall	-	13	98	0.29	0.59	0.95	0.99	0.21	0.54	1.08	2.58	0.24	0.53	1.02	1.24
50% survival quantile	Random	20/arm	3	20	0.41	0.60	0.79	1.00	0.31	0.46	0.65	0.68	0.31	0.49	0.68	0.70
		50/arm	3	16	0.31	0.64	1.07	1.02	0.35	0.79	1.12	7.71	0.42	0.79	1.15	0.99
		100/arm	3	20	0.70	0.88	1.28	1.29	0.80	0.98	1.20	1.76	0.75	0.94	1.20	1.15
		200/arm	3	16	0.42	0.84	1.02	0.94	0.52	0.67	1.16	1.05	0.48	0.67	1.22	1.12
	TCGA	200 total	1	18	0.16	0.30	0.53	0.57	0.07	0.21	0.29	0.42	0.08	0.22	0.54	6.96
	Overall	-	13	90	0.34	0.62	0.96	1.00	0.30	0.58	1.08	3.41	0.34	0.63	1.14	3.24
25% survival quantile	Random	20/arm	3	17	0.19	0.40	1.22	1.14	0.34	2.43	58.71	63.88	0.27	0.35	0.55	0.79
		50/arm	3	13	0.78	0.92	1.12	1.07	0.59	0.79	1.21	15.65	0.66	0.80	1.04	1.00
		100/arm	3	17	0.50	0.95	1.08	1.03	0.46	0.78	4.30	7.48	0.43	0.78	1.01	0.88
		200/arm	3	12	0.23	0.51	1.04	0.98	0.51	0.79	1.93	4.66	0.36	0.53	1.07	1.11
	TCGA	200 total	1	9	0.12	0.55	0.71	0.63	0.08	0.11	0.35	0.38	<0.01	0.11	0.50	27.97
	Overall	-	13	68	0.38	0.64	1.09	1.02	0.34	0.78	3.74	32.94	0.29	0.54	0.95	10.21

Metric	Dataset(s)		No. distinct figures	No. IPD sets	Errors by IPD reconstruction method (unit: 10^{-3})											
					Direct				Guyot				Rogula			
	Type	Size			25% AE	50% AE	75% AE	RMSE	25% AE	50% AE	75% AE	RMSE	25% AE	50% AE	75% AE	RMSE
Survival at t=0.6	Random	20/arm	3	20	0.00	0.00	0.00	0.00	0.00	3.02	18.26	16.71	0.00	0.00	0.00	0.00
		50/arm	3	20	0.00	0.00	0.00	0.03	1.13	2.08	6.64	11.27	0.00	0.00	0.00	0.05
		100/arm	3	20	0.00	0.00	0.00	0.07	0.63	1.06	1.54	2.26	0.00	0.00	0.05	1.01
		200/arm	3	20	<0.01	0.04	0.15	1.00	0.88	1.09	1.63	2.10	0.01	0.08	0.98	1.94
	TCGA	200 total	1	18	0.00	0.00	0.00	0.14	1.55	1.96	4.00	3.15	0.00	0.00	0.98	2.29
	Overall	-	13	98	0.00	0.00	0.00	0.46	0.83	1.49	4.00	9.31	0.00	0.00	0.04	1.39
Survival at t=0.8	Random	20/arm	3	20	0.00	0.00	0.00	19.36	0.00	12.97	22.33	21.33	0.00	0.00	0.00	15.81
		50/arm	3	20	0.00	0.00	0.00	0.03	1.13	1.78	5.10	4.06	0.00	0.00	0.00	0.05
		100/arm	3	20	0.00	0.00	0.00	0.06	0.39	0.78	1.85	2.00	0.00	0.00	0.02	0.66
		200/arm	3	20	<0.01	0.02	0.24	0.82	0.74	1.27	1.88	1.50	<0.01	0.02	0.95	1.43
	TCGA	200 total	1	18	0.00	0.00	0.00	0.14	1.55	1.96	4.00	3.15	0.00	0.00	0.98	2.29
	Overall	-	13	98	0.00	0.00	<0.01	8.76	0.82	1.55	4.40	9.97	0.00	0.00	0.02	7.25
Survival at t=1.0	Random	20/arm	3	20	0.00	0.00	0.00	11.80	0.00	7.92	17.94	12.93	0.00	0.00	0.00	0.00
		50/arm	3	20	0.00	0.00	0.00	0.02	3.28	4.87	6.49	5.42	0.00	0.00	0.00	0.04
		100/arm	3	20	0.00	0.00	0.00	0.05	0.20	0.31	0.99	1.58	0.00	0.00	0.01	0.53
		200/arm	3	20	0.00	0.03	0.36	1.49	0.36	0.81	1.00	0.91	<0.01	0.01	1.48	1.61
	TCGA	200 total	1	18	0.00	0.00	0.00	0.14	1.55	1.96	4.00	3.15	0.00	0.00	0.98	2.29
	Overall	-	13	98	0.00	0.00	0.00	5.37	0.36	1.53	4.53	6.53	0.00	0.00	0.01	1.25

Suppl. Table 2

Survival metrics including hazard ratios between cohorts of a figure and the area between the Kaplan-Meier curves per cohort were calculated. Exponential survival regression was performed per cohort and its regression parameter (lambda) was obtained. Landmark survival and survival quantiles were calculated within cohorts. Absolute errors (unit: 10^{-3}) between metrics based on the reconstructed and the original datasets were calculated and their ***means*** *(MAE) and standard deviations (SD) are reported.*

Metric	Dataset(s)		No. distinct figures	No. IPD sets	Errors by method (unit: 10^{-3})					
					Direct		Guyot		Rogula	
	Type	Size			MAE	SD	MAE	SD	MAE	SD
Hazard ratio	Random	20/arm	3	10	9.78	29.95	45.90	38.80	0.19	0.39
		50/arm	3	10	1.27	1.65	22.02	23.43	0.51	0.81
		100/arm	3	10	0.49	0.38	10.14	13.27	0.60	0.86
		200/arm	3	10	6.85	10.74	2.45	1.33	6.92	6.71
	Real-world (TCGA)	200 total	1	9	2.04	2.30	6.65	2.12	11.53	16.78
	Overall	-	13	49	4.13	14.31	17.65	25.93	3.79	8.72
Log hazard ratio	Random	20/arm	3	10	5.44	11.36	35.14	23.73	1.17	2.47
		50/arm	3	10	1.89	4.13	22.56	21.31	0.41	0.41
		100/arm	3	10	0.49	0.28	7.22	7.10	0.53	0.53
		200/arm	3	10	3.54	3.84	2.61	2.30	4.18	2.58
	Real-world (TCGA)	200 total	1	9	0.77	0.86	2.50	0.80	4.35	6.35
	Overall	-	13	49	2.46	5.81	14.24	19.26	2.08	3.51
SE log HR	Random	20/arm	3	10	0.77	2.17	6.15	7.03	0.05	0.11
		50/arm	3	10	0.07	0.18	0.91	0.91	<0.01	<0.01
		100/arm	3	10	<0.01	<0.01	0.42	0.58	0.06	0.16
		200/arm	3	10	0.08	0.08	0.10	0.14	0.07	0.08
	Real-world (TCGA)	200 total	1	9	0.01	0.02	0.14	0.03	0.14	0.28
	Overall	-	13	49	0.19	0.99	1.57	3.88	0.06	0.15
Area between the curve	Random	20/arm	3	20	0.94	0.86	7.28	6.56	0.87	0.73
		50/arm	3	20	0.62	0.29	2.97	1.37	0.60	0.28
		100/arm	3	20	0.71	0.57	1.82	1.21	0.87	0.78
		200/arm	3	20	0.97	0.70	1.66	0.63	1.33	0.84

Metric	Dataset(s)		No. distinct figures	No. IPD sets	Errors by method (unit: 10^{-3})					
					Direct		Guyot		Rogula	
	Type	Size			MAE	SD	MAE	SD	MAE	SD
	Real-world (TCGA)	200 total	1	18	0.36	0.34	2.34	1.00	1.22	1.80
	Overall	-	13	98	0.73	0.63	3.23	3.72	0.97	1.01
Exponential survival regression (lambda)	Random	20/arm	3	20	6.36	19.17	15.54	16.80	1.91	1.78
		50/arm	3	20	1.27	1.01	18.06	17.10	1.31	1.13
		100/arm	3	20	2.34	3.04	4.47	5.07	2.22	2.82
		200/arm	3	20	2.80	2.39	2.36	1.34	2.56	1.71
	Real-world (TCGA)	200 total	1	18	1.29	1.95	27.53	1.58	8.45	15.71
	Overall	-	13	98	2.84	8.91	13.31	14.23	3.18	7.26
75% survival quantile	Random	20/arm	3	20	0.93	0.78	0.87	0.73	0.88	0.73
		50/arm	3	20	0.66	0.57	2.07	5.06	0.63	0.55
		100/arm	3	20	0.94	0.87	1.06	0.79	0.84	0.79
		200/arm	3	20	0.68	0.48	0.67	0.51	0.91	0.98
	Real-world (TCGA)	200 total	1	18	0.43	0.48	0.35	0.45	0.81	1.51
	Overall	-	13	98	0.73	0.67	1.02	2.38	0.81	0.94
50% survival quantile (median)	Random	20/arm	3	20	0.76	0.67	0.57	0.39	0.59	0.39
		50/arm	3	16	0.78	0.68	3.17	7.26	0.83	0.56
		100/arm	3	20	1.09	0.70	1.34	1.16	1.05	0.50
		200/arm	3	16	0.81	0.51	0.91	0.53	0.95	0.63
	Real-world (TCGA)	200 total	1	18	0.41	0.40	0.28	0.33	2.51	6.68
	Overall	-	13	90	0.78	0.63	1.21	3.20	1.18	3.03
25% survival quantile	Random	20/arm	3	17	0.79	0.85	41.84	49.76	0.56	0.57
		50/arm	3	13	0.98	0.45	6.78	14.69	0.86	0.54
		100/arm	3	17	0.88	0.56	3.96	6.54	0.76	0.45
		200/arm	3	12	0.73	0.69	2.61	4.03	0.87	0.72
	Real-world (TCGA)	200 total	1	9	0.49	0.41	0.25	0.30	9.51	27.90
	Overall	-	13	68	0.80	0.63	13.24	30.38	1.91	10.11
	Random	20/arm	3	20	0.00	0.00	10.38	13.43	0.00	0.00

Metric	Dataset(s)		No. distinct figures	No. IPD sets	Errors by method (unit: 10^{-3})					
					Direct		Guyot		Rogula	
	Type	Size			MAE	SD	MAE	SD	MAE	SD
Survival at t=0.6		50/arm	3	20	<0.01	0.03	5.35	10.18	0.02	0.05
		100/arm	3	20	0.02	0.06	1.54	1.70	0.26	1.01
		200/arm	3	20	0.42	0.93	1.61	1.38	0.96	1.72
	Real-world (TCGA)	200 total	1	18	0.04	0.14	2.77	1.54	1.00	2.13
	Overall	-	13	98	0.10	0.45	4.36	8.27	0.44	1.33
Survival at t=0.8	Random	20/arm	3	20	7.50	18.32	15.24	15.31	5.00	15.39
		50/arm	3	20	<0.01	0.03	3.14	2.65	0.02	0.05
		100/arm	3	20	0.01	0.06	1.26	1.58	0.17	0.66
		200/arm	3	20	0.37	0.75	1.30	0.76	0.70	1.28
	Real-world (TCGA)	200 total	1	18	0.04	0.14	2.77	1.54	1.00	2.13
	Overall	-	13	98	1.62	8.65	4.78	8.79	1.38	7.15
Survival at t=1.0	Random	20/arm	3	20	2.64	11.80	9.06	9.47	0.00	0.00
		50/arm	3	20	<0.01	0.02	5.02	2.10	0.01	0.04
		100/arm	3	20	0.01	0.05	0.91	1.33	0.14	0.52
		200/arm	3	20	0.58	1.41	0.76	0.53	0.88	1.39
	Real-world (TCGA)	200 total	1	18	0.04	0.14	2.77	1.54	1.00	2.13
	Overall	-	13	98	0.67	5.36	3.72	5.39	0.39	1.19
