# Supplementary material for: Visual extraction of individual patient data from published kaplan-meier survival plots using a web-based application offering different reconstruction methods (V-Exploder)
Source: BMC Med Res Methodol. 2026 Oct 5;26:216. doi: 10.1186/s12874-026-03036-3 (PMC13640098; doi:10.1186/s12874-026-03036-3)
Supplement: Supplementary file 2 [file 12874_2026_3036_MOESM2_ESM.zip › v-exploder-source/dist/index.html]

V-Exploder
