# Supplementary material for: Visual extraction of individual patient data from published kaplan-meier survival plots using a web-based application offering different reconstruction methods (V-Exploder)
Source: BMC Med Res Methodol. 2026 Oct 5;26:216. doi: 10.1186/s12874-026-03036-3 (PMC13640098; doi:10.1186/s12874-026-03036-3)
Supplement: Supplementary file 2 [file 12874_2026_3036_MOESM2_ESM.zip › v-exploder-source/src/docs/about.html]

V-Exploder is a web-based application facilitating the visual extraction of
individual patient-level survival data from published Kaplan-Meier plots.
Visit the website
for more information or read the
documentation
to learn more about data extraction and to get started.

This software is provided free of charge "as is". The accuracy of
any extracted data depends largely on the image quality, the nature of the
data and the user himself/herself. Thus, no guarantee can be given for the
correctness of derived data. By using V-Exploder, you agree to the
terms of use.

This web application is developed by  
Dr. Fabian Acker  
Goethe University Frankfurt, University Hospital  
Department of Internal Medicine II, Hematology and Oncology  
Theodor-Stern-Kai 7  
60590 Frankfurt/Main  
Germany  
E-Mail: mail@v-exploder.com

I am very happy to receive feedback and suggestions.
